# Transition of Hypertriglyceridemic-Waist Phenotypes and the Risk of Type 2 Diabetes Mellitus among Middle-Aged and Older Chinese: A National Cohort Study

**DOI:** 10.3390/ijerph18073664

**Published:** 2021-04-01

**Authors:** Ge Chen, Qian Yi, Leying Hou, Shenghan Peng, Mengya Fan, Peige Song, Yimin Zhu

**Affiliations:** 1 Medical Research and Biometrics Center, National Center for Cardiovascular Diseases, Beijing 102300, China; chenge@mrbc-nccd.com; 2 School of Public Health, Zhejiang University School of Medicine, Zhejiang University, Hangzhou 310058, China; qianyizj@zju.edu.cn (Q.Y.); haddyhou@zju.edu.cn (L.H.); 3170103881@zju.edu.cn (M.F.); zhuym@zju.edu.cn (Y.Z.); 3 School of Public Health, The University of Edinburgh, Edinburgh EH8 9SU, UK; s1923751@ed.ac.uk; 4 Women’s Hospital, Zhejiang University School of Medicine, Hangzhou 310058, China

**Keywords:** hypertriglyceridemic-waist, type 2 diabetes mellitus, risk factors, transition, China

## Abstract

The rapid economic growth and nutritional changes in China have brought an increased burden of type 2 diabetes mellitus (T2DM). This study aimed to assess the effects of hypertriglyceridemic-waist (HTW) and its dynamic transitions on incident T2DM among middle-aged and older Chinese. Data were extracted from the China Health and Retirement Longitudinal Study (CHARLS). Participants were classified into three HTW phenotypes, namely NTNW (normal triglyceride (TG) and waist circumference (WC)), NTEW/ETNW (normal TG and enlarged WC, or elevated TG and normal WC) and ETEW (elevated TG and enlarged WC). Multivariable Cox frailty models were used to assess the associations of HTW phenotypes and their transitions over time with the risk of T2DM. A total of 7397 subjects without T2DM were included, of which 849 developed T2DM during 2011–2018. Compared with individuals with NTNW, people in the NTEW/ETNW group and ETEW group were at a significantly higher risk of T2DM (HR_NTEW/ETNW_ = 1.28, 95% CI: 1.06–1.54 and HR_ETEW_ = 1.61, 95% CI: 1.26–2.06). For subjects with NTNW at baseline, the risk of developing T2DM increased by 38% and 83% if their metabolic status changed to NTEW/ETNW and ETEW, respectively. For subjects with NTEW/ETNW, the risk of T2DM decreased by 33% when their metabolic status changed to normal (NTNW); but the risk increased by 49% if the status became more serious (ETEW). NTEW/ETNW, ETEW and their transitions to adverse states were risk factors for T2DM.

## 1. Introduction

Diabetes is a highly prevalent metabolic disease characterized by dysfunctional glucose regulation, and the predominant form is type 2 diabetes mellitus (T2DM) [1]. T2DM is an important cause of mortality and disability and was estimated to affect 422 million individuals worldwide in 2014 [2]. In addition, the burden of T2DM in low- and middle-income countries (LMICs) has increased during the past few decades, especially in China and India [3,4]. The prevalence of T2DM in Chinese adults has increased sharply from 4.5% in 1995–1999 to 8.3% in 2010–2014 [5]. It is estimated that by 2025, the prevalence of T2DM in China will reach 12.5%, placing a considerable threat to families and the Chinese society [6].

Hypertriglyceridemic-waist (HTW) is a comprehensive metabolic indicator, which includes the presence of enlarged waist circumference (WC) and elevated triglyceride (TG) levels. The concept of HTW was first proposed as an index for cardiovascular disease by Lemieux et al. in the late twentieth century [7]. Since then, pathophysiologic and epidemiologic evidence revealed associations between HTW and elevated insulin resistance and excessive activation of β-cell function [8,9]. Given that HTW is essential for the identification and early prevention of insulin resistance, it has been proposed as an inexpensive and reliable screening tool for T2DM.

HTW is a transient state of health. Previous research has demonstrated a positive link between HTW and T2DM, whereas studies on the impacts of different HTW transition patterns on T2DM were limited in China. Early identification of HTW transitions patterns is of significant importance in preventing the development of T2DM [10]. In this study, we aimed to explore the associations between HTW phenotypes and new-onset T2DM, and to assess the effect of dynamic HTW transitions on the development of T2DM among middle-aged and older Chinese.

## 2. Materials and Methods

### 2.1. Study Design and Study Population

Data were extracted from the China Health and Retirement Longitudinal Study (CHARLS, available at http://charls.pku.edu.cn/en, accessed on 1 August 2020). CHARLS is an ongoing nationwide and household-based survey in Chinese middle-aged and older adults, starting in 2011 and being followed up every 1–2 years [11,12]. So far, CHARLS has been conducted for five rounds (in 2011, 2013, 2014, 2015 and 2018 respectively), among which the 2011 round (baseline) and the 2015 round provided biomarker data, and the 2014 round is the Life History Survey. Details about the study design and implement procedures of CHARLS are available elsewhere [11,12,13]. Briefly, CHARLS was conducted using a probability-proportional-to-size sampling strategy (PPS) and covered 28 provinces across Mainland China (locations of the investigated provinces are listed in Figure S1 and Table S1). First, all counties in invited provinces were stratified by regions, urban/rural settings and economic status, among which 150 counties were randomly selected. Then, three primary sampling units (PSU), administrative villages in rural settings and communities in urban settings, were randomly chosen from each county. Third, at least 24 households within each PSU were mapped using “CHARLS-GIS” software for participation, from which one individual aged 45 years or above, together with his/her spouse were interviewed.

**Figure 1:**
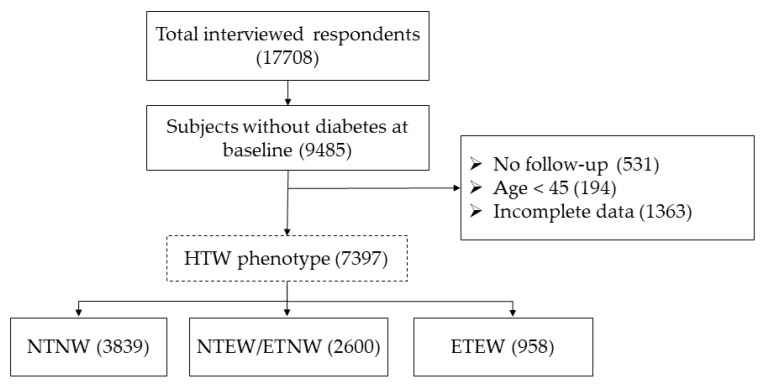
Flow chart of subjects included in this study. Note: Incomplete data referred to incomplete information on demographics (age and sex), socioeconomics (educational attainment and marital status), geography (setting and geographic region) and anthropometric measurements.

In this national cohort study, 17,708 participants with a household response rate of 80.5% from four CHARLS rounds (2011, 2013, 2015 and 2018) were obtained. Individuals aged ≥ 45 years, with completed demographic, socioeconomic, geographic, anthropometric and biomarker data, and free of diabetes at baseline (in 2011) were included for analyses. We excluded subjects who were diagnosed as T2DM at baseline (*n* = 8223), did not respond to the follow-up (*n* = 531), aged < 45 years (*n* = 194), and without complete information of demographics, socioeconomics, geography and anthropometric measurements at baseline (*n* = 1363). Finally, a total of 7397 subjects were included in our cohort study. The selection procedure is summarized in Figure 1.

Ethical approval for the study was granted by the Ethical Review Committee of Peking University and conducted by the National School for Development (China Centre for Economic Research) at Peking University following the Helsinki guideline. The Institutional Review Board (IRB) approval number for the main household survey, including anthropometrics, is IRB00001052-11015; the IRB approval number for biomarker collection, was IRB00001052-11014. All participants signed written informed consent forms.

### 2.2. Data Collection

From CHARLS 2011 to 2018, trained interviewers collected information on demographics, socioeconomic status, geographic location, health-related behaviors and medical history by a structured questionnaire. Anthropometric measurements were taken following a standard protocol from the World Health Organization [11]. Body weight was measured to the nearest 0.1 kg in lightweight clothes and without shoes by digital weight scale (OMRON Corporation, HN-286). Body height was measured to the nearest 0.1 centimeters without shoes on a stadiometer (Seca Corporation, 213). WC was horizontally measured at the midpoint of the line between the lower rib and the upper iliac crest without coats by soft measure tape. Using an electronic sphygmomanometer (OMRON Corporation, HEM-7200), blood pressure (BP) was measured on the participant’s right arm three times separated by a 45-s intervals. Systolic blood pressure (SBP) and diastolic blood pressure (DBP) were respectively recorded as the average of the three readings. Body mass index (BMI) was calculated as body weight divided by squared height (kg/m^2^).

Venous blood samples were collected from participants after at least 12 h of overnight fasting by professional staffs. The complete blood count was conducted within 1–2 h after blood collection at the survey sites. Then the specimen of whole blood was stored at 4 °C for later testing of glycated hemoglobin (HbA1c) and the remaining samples were transported back to the central Laboratory in Beijing (Youanmen Centre for Clinical Laboratory of Capital Medical University) for further laboratory analyses [11,13]. The glucose, triglyceride, total cholesterol (TC), high-density lipoprotein cholesterol (HDL-C), and low-density lipoprotein cholesterol (LDL-C) levels were measured with an enzymatic-colorimetric test. The HbA1c levels were tested using a boronate-affinity high-performance liquid chromatography method [13].

### 2.3. Definitions of HTW Phenotypes and T2DM

In accordance with the definition criteria from International Diabetes Federation, participants in this study were classified into three HTW phenotypes, namely NTNW, NTEW/ETNW, and ETEW [14]. According to the transition patterns of three HTW phenotypes during follow-up, nine transition subgroups were defined. Definitions of the three HTW phenotypes and the nine transition groups (A-I) are listed in Table 1.

The primary definition of T2DM was set based on the World Health Organization (WHO) recommendation [15]. An individual was considered as having T2DM if his/her ≥126 mg/dL (7.0 mmol/L), and/or random blood glucose ≥200 mg/dL (11.1 mmol/L), and/or HbA1c ≥ 6.5%, and/or self-reported physician diagnosis through the question “Have you been diagnosed with diabetes or hyperglycemia?”, and/or currently under antihyperglycemic treatment, including traditional Chinese and modern Western medicine, and/or under other treatments [15].

### 2.4. Definitions of Covariates

We used information on age (45–49 years, 50–59 years, 60–69 years, and 70 years and above), sex (male and female), education (illiterate, literate, primary education, and middle school education and above), and marital status (married or cohabiting and single). For the assessment of household economic status, tertiles of the natural logarithm of per capita expenditures (ln (PCE)) were adopted as an indicator of family wealth [16]. The bottom, middle and top tertiles respectively represented the poor, middle and rich status. Regarding the geographic characteristics, we classified participants’ residence into North China, Northeast China, East China, South-central China, Southwest China and Northwest China (see Supplementary Figure S1 and Table S1 for more details). In addition, well-established health behaviors, i.e., smoking and alcohol drinking, were included in our analysis. Participants were classified as smokers and non-smokers by the question “did you smoke at least one cigarette per day last year?” [17]. Similarly, people who drank at least once per week in the last year were identified as drinkers based on a face-to-face interview. Following the Working Group on Obesity in China (WGOC) criteria, overweight was defined as a BMI ≥ 24 kg/m^2^ and obesity as a BMI ≥ 28 kg/m^2^ [18]. Hypertension was defined by an SBP ≥ 140 mmHg, and/or a DBP ≥ 90 mmHg, and/or a self-reported physician diagnosis, and/or currently with antihypertensive drugs, and/or under other related therapeutic measures [19].

### 2.5. Statistical Analysis

The basic characteristics of the included participants were reported using percentages for categorical variables. The comparison of basic characteristics between urban and rural settings was performed by Chi-square test. Person-years was calculated from the date of baseline interview and anthropometric measurements (CHARLS 2011) until the occurrence of T2DM events or the end of follow-up (CHARLS 2018), whichever came first. The cumulative incidence rates of T2DM were calculated by the Kaplan–Meier method. We also compared the differences in T2DM cumulative incidence among HTW phenotype using the Log rank test.

The hazard ratios (HRs) for incident T2DM by HTW phenotype were calculated using multivariable Cox frailty models with random intercepts to account for clustering of participants by city. The phenotype NTNW was used as a referent category. Three core models were conducted as follows: Model 1 adjusted for age and sex. Then, Model 2 additionally adjusted for education, marital status, ln (PCE) by setting, region, obesity, smoking, and drinking based on Model 1. Finally, Model 3 additionally adjusted for all the variables in Model 2 plus SBP, DBP, TC, HDL-C, and LDL-C levels. In addition, three consistent groups (group A, E and I) were set as references, compared to which HRs for group B and C, D and F, G and H were calculated by multivariable Cox frailty models. Covariates including age, sex, education, region, obesity, smoking, drinking, SBP, DBP, TC, HDL-C and LDL-C level, were adjusted for.

All analyses were conducted in SAS statistical software (version 9.4; SAS Institute Inc., Cary, NC, USA). A *p* value of less than 0.05 was indicative of statistical significance. All tests were two-sided.

## 3. Results

### 3.1. Characteristics of Study Subjects

In CHARLS 2011, a total of 7397 subjects without T2DM were included out of 17,311 study participants. We compared the general characteristics of included (*n* = 7397) and excluded subjects (*n* = 9914) (Table S2). Table 2 shows the demographic, socioeconomic and geographic characteristics, health behaviors and HTW phenotypes in rural and urban settings. Approximately one-third of participants were aged 50–59 years (35.7%) and 60–69 years (29.5%). The proportion of females (53.4%) was a bit higher than that of males (46.6%). Just under 30% of participants were illiterate (29.4%) or had received middle or higher education (29.2%). The majority of participants were married or cohabiting, middle-income and East China dwellers. In addition, there were significant differences between rural and urban residents in terms of educational achievement, economic level, geographical location, HTW phenotype, nutritional status and lifestyle.

### 3.2. Cumulative T2DM Incidence

**Figure 2:**
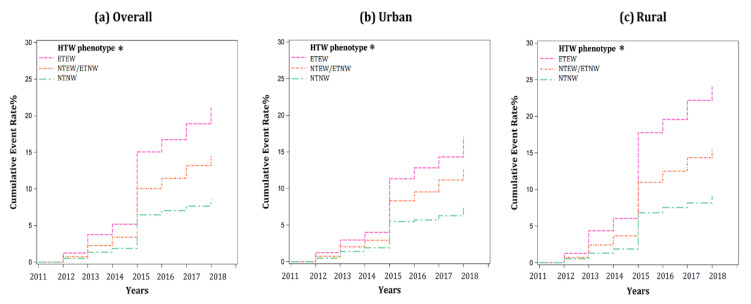
Cumulative incidence of T2DM for the three HTW phenotypes (NTNW, NTEW/ETNW and ETEW) from CHARLS 2011 to 2018.Note: (**a**) Overall, (**b**) Urban, (**c**) Rural; NTNW, normal TG level and normal WC; NTEW, normal TG level and enlarged WC; ETNW, elevated TG level and normal WC; ETEW, elevated TG level and enlarged WC. HTW phenotypes *, the cumulative rate of T2DM is different across three HTW phenotypes in urban, rural and total participants.

In CHARLS, from 2011 to 2018, a total of 849 participants aged 45 years or above developed T2DM, of which 605 were rural dwellers. The cumulative incidence rate of T2DM in rural areas (12.9%) was significantly higher than that in urban areas (11.0%). The cumulative incidence of T2DM increased steadily each year, and a sharp increase was observed in CHARLS 2015 (Figure 2a). When stratified by the three HTW phenotype groups, significant differences were observed in the cumulative rates of T2DM in urban (7.3%, 12.5% and 17.0%, respectively) and rural settings (9.1%, 15.4% and 24.0%, respectively) (Figure 2b,c). Participants with ETEW were the most likely to develop T2DM, and the NTNW group was at the lowest risk.

### 3.3. Risk of Incident T2DM by HTW Phenotype

Table 3 presents the HRs for developing T2DM across the three HTW phenotypes. Compared with individuals with NTNW, people in the NTEW/ETNW group and ETEW group were at a significantly higher risk of T2DM, with crude HRs of 1.69 (95% CI: 1.45, 1.97) and 2.58 (95% CI: 2.15, 3.10), respectively. In age- and sex-adjusted models, the overall risk of T2DM was markedly elevated (HR_NTEW/ETNW_ = 1.68, 95% CI: 1.44–1.97 and HR_ETEW_ = 2.54, 95% CI: 2.11–3.07). Additional adjustments for education, marital status, ln(PCE) by setting, region, obesity, smoking and drinking did not substantially change the associations (HR_NTEW/ETNW_ = 1.39, 95% CI: 1.16–1.66 and HR_ETEW_ = 1.88, 95% CI: 1.50–2.36). Similar findings were observed after further adjusting for SBP, DBP, TC, HDL-C and LDL-C (HR_NTEW/ETNW_ = 1.28, 95% CI: 1.06–1.54 and HR_ETEW_ = 1.61, 95% CI: 1.26–2.06). The associations between HTW phenotypes and T2DM in urban and rural settings followed similar risk patterns. The HRs for T2DM by potential confounders are described in more detail in Table S3.

### 3.4. Risk of Incident T2DM by HTW Transition

**Figure 3:**
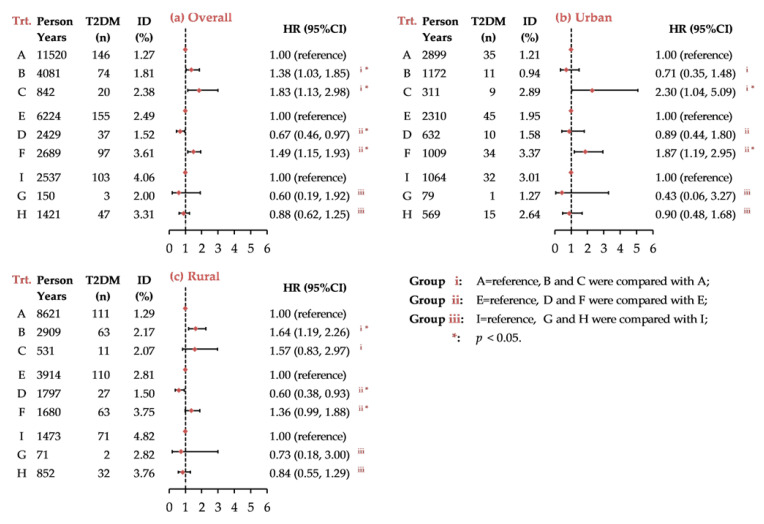
Risk of incident T2DM by transition of HTW phenotypes in middle-aged and older Chinese. Note: Data were presented as *n* (%) and hazard ratio (95% CI), adjusted for age, sex, education, region, obesity, smoking, drinking, SBP, DBP, TC, HDL-C level and LDL-C level; ID, incidence density; Trt., transition during follow-up, the definitions of group A to I were listed as follows: Group A, the consistent phenotype of NTNW (reference); Group B, NTNW to NTEW/ETNW; Group C, NTNW to ETEW; Group E, the consistent phenotype of NTEW/ETNW (reference); Group D, NTEW/ETNW to NTNW; Group F, NTEW/ETNW to ETEW; Group I, the consistent phenotype of ETEW (reference); Group G, ETEW to NTNW; Group H, ETEW to NTEW/ETNW.

The characteristics of the nine transition groups are shown in Supplemental Table S4. The multivariable-adjusted HRs of incident T2DM stratified by nine transition patterns are profiled in Figure 3. Groups A, E, and I are the consistent transition phenotypes (maintaining NTNW, NTEW/ETNW and ETEW during follow-up, respectively). Overall, participants in group B (NTNW changed to NTEW/ETNW, HR = 1.38, 95% CI: 1.03–1.85) and C (NTNW changed to ETEW, HR = 1.83, 95% CI: 1.13–2.98) were at a significantly higher risk of T2DM than those in group A. Compared with participants in group E, group F participants (NTEW/ETNW changed to ETEW) were more likely to develop T2DM (HR = 1.49, 95% CI: 1.15–1.93), but those in group D (NTEW/ETNW changed to NTNW) were at a lower risk (HR = 0.67, 95% CI: 0.46–0.97). For participants with ETEW at baseline (group I, G and H), no significant differences were observed between different transitions. There were generally similar patterns for participants in urban (HR_C vs. A_ = 2.30, 95% CI: 1.04–5.09 and HR_F vs. E_ = 1.87, 95% CI: 1.19–2.95) and rural settings (HR_B vs. A_ = 1.64, 95% CI: 1.19–2.26, HR_D vs. E_ = 0.60, 95% CI: 0.38–0.93 and HR_F vs. E_ = 1.36, 95% CI: 0.99–1.88).

## 4. Discussion

In this study, the cumulative incidence of T2DM was 12.9% and 11.0% for urban and rural Chinese residents, respectively, during a median of 7 years follow-up (2011–2018). People with NTEW/ETNW were 1.28-fold as likely to develop T2DM as those with normal WC and TG levels (NTNW). Our study provides evidence that ETEW is a major metabolic risk for T2DM among middle-aged and older Chinese people (HR = 1.61) regardless of residence. For subjects with NTNW at baseline, the risk of T2DM could increase by 38% and 83% when the state of NTNW changed to unhealthy metabolic status (NTEW/ETNW and ETEW); for subjects with NTEW/ETNW, the risk of T2DM decreased by 33% when the state of NTEW/ETNW changed to normal (NTNW), and increased by 49% when the state of NTEW/ETNW became more serious (ETEW). However, if people had already developed ETEW, the risk of T2DM did not decrease and even ETEW became a less serious state during follow-up.

In our study, individuals in rural areas were at a higher risk of developing T2DM than those in urban residents, which differs from findings in previous studies [20]. Since the Reform and Opening in the late 1970s, the Chinese economy has developed rapidly. Sedentary lifestyles and dietary changes to Western patterns have resulted in a high prevalence of chronic non-communicable diseases over the past decade [21]. In an investigation conducted by the China Kadoorie Biobank Collaborative Group, rural areas had higher rates of diabetes-related mortality, despite the fact that diabetes was more prevalent in urban areas [22]. Furthermore, in a study conducted in Zhejiang province, prevalence of T2DM increased rapidly between 2007 and 2017, and the average annual increase was higher in rural regions than in urban regions [23]. Additionally, evidence from a large-scale epidemiological study, which noted that BMI was increasing at the same rate or faster in rural areas compared with urban areas in LMICs, may partly explain our results [24]. Those findings indicate the necessity of promoting healthy lifestyles (e.g., healthy diet, exercise) and T2DM screening for Chinese rural residents to prevent and manage this disease.

A large number of investigations have suggested that central obesity and hypertriglyceridemia are independent risk factors for dysfunctional glucose regulation [25,26,27,28]. Our study extends the existing knowledge by demonstrating associations of a specific metabolic abnormality—HTW phenotype—with T2DM in a nationwide prospective cohort study. Compared with NTNW subjects, subjects with NTEW/ETNW had a higher risk of abnormal glucose metabolism and this risk could be further increased in participants with ETEW. These findings are largely consistent with a previous study conducted in Isfahan, which reported that individuals with very high WC and TG levels were more likely to develop T2DM compared with the NTNW group in a population aged 30–70 years [29]. According to a Chinese study in a rural setting in Henan, the HTW phenotype was associated with T2DM, with a 1.59-fold risk of T2DM in NTEW/ETNW group and a 2.10-fold risk in ETEW patients, which is comparable to our estimate (HR_NTEW/ETNW_ = 1.28 and HR_ETEW_ = 1.61) [30]. Despite the different geographical and socio-economic contexts, the significant associations highlight the importance of this simple and inexpensive indicator in screening for individuals at risk of abnormal glucose metabolism.

Previous studies have emphasized the importance of HTW phenotypes [31,32,33]; however, not many investigations have focused on the effects of HTW transitions on T2DM. In the current study, we compared the risk of T2DM in participants with dynamic HTW transitions with that of their counterparts with consistent HTW phenotypes over time. For people with normal WC and TG levels (NTNW) at baseline, the association between HTW phenotype and T2DM risk was strengthened with the severity of adverse transitions. For individuals with NTEW/ETNW at baseline, the transition to normal lipid metabolism resulted in a lower risk (HR = 0.67) of T2DM, whereas the risk increased by 49% when NTEW/ETNW transitioned to ETEW. However, this effect seems to be irreversible for participants who developed ETEW at baseline. Compared with the ETEW consistent group, no significant differences were witnessed for participants whose WC and TG levels decreased during follow-up.

The mechanism of how various HTW phenotypes influence T2DM is still unclear. It is reported that the central distribution of body fat, especially the accumulation of visceral adipose tissue (VAT), triggers an immune response and leads to an excessive amount of free fatty acids (FFA) [34]. The highly concentrated FFA in portal vein circulation induces insulin resistance and inhibits the effects of glucose uptake [35]. Although the increase in insulin secretion temporarily compensates for these changes, persistently high glucose levels may contribute to the development of T2DM [36]. Moreover, the immune response caused by VAT and high TG levels, which differs from other types of inflammation, could be long-lasting and irreversible in some cases [36]. Such a mechanism suggests that efforts such as improving the awareness of central obesity and promoting healthy diets and regular exercise, are needed to prevent early-onset of T2DM, especially in slightly obese adults (NTNW and NTEW/ETNW) in China.

The current study, to the best of our knowledge, serves as the first investigation that evaluated the associations of HTW transitions with incident T2DM at the national level. With a large geographic coverage (28 out of 31 provinces) and a large number of samples, the data from the study were relatively robust. Another prominent feature of this study was the adoption of PCE as a surrogate of household resources, which, in developing countries, is a better welfare indicator than gross domestic product per capita [37]. Furthermore, all blood specimens were tested using a standard protocol in a single laboratory, largely reducing potential biases arising from the use of different methodologies.

However, this study is not without its limitations. First, our study included only middle-aged and older Chinese people, therefore the relationships between HTW phenotype, transitions and incident T2DM in young adults could not be explored. Second, due to the original design of CHARLS, blood sample tests were only carried out in 2011 and 2015, which led to an apparent sudden increase in T2DM incidence in 2015. Third, we only explored the risk of T2DM adjusting for a limited number of variables in the multivariable Cox frailty models; other confounding factors, such as dietary patterns, exercise habits, and family history of T2DM, were not included due to the absence of relevant information.

## 5. Conclusions

In this study, NTEW/ETNW, ETEW and their transitions to adverse states were risk factors for T2DM. Efforts are needed to prevent the onset of T2DM among people with hypertriglyceridemia or central obesity.

## Figures and Tables

**Table 1:** Definitions of the three HTW phenotypes and the nine transition groups.

HTW Phenotypes	Definition
NTNW	Normal TG level (≤150 mg/dL (1.7 mmol/L)) and Normal WC (<90 cm in males or <85 cm in females)
NTEW/ETNW	Normal TG level (≤150 mg/dL (1.7 mmol/L)) and Enlarged WC (≥90 cm in males or ≥85 cm in females); or Elevated TG level (>150 mg/dL (1.7 mmol/L)) and Normal WC (<90 cm in males or <85 cm in females)
ETEW	Elevated TG level (>150 mg/dL (1.7 mmol/L)) and Enlarged WC (≥90 cm in males or ≥85 cm in females)
**Transition Groups**	**Definition**
A	The consistent phenotype groups of NTNW during follow-up
B	NTNW at baseline changed to NTEW/ETNW at follow-up
C	NTNW at baseline changed to ETEW at follow-up
D	NTEW/ETNW at baseline changed to NTNW at follow-up
E	The consistent phenotype groups of NTEW/ETNW during follow-up
F	NTEW/ETNW at baseline changed to ETEW at follow-up
G	ETEW at baseline changed to NTNW at follow-up
H	ETEW at baseline changed to NTEW/ETNW at follow-up
I	The consistent phenotype groups of ETEW during follow-up

Note: HTW, Hypertriglyceridemic–waist; TG, triglyceride; WC, waist circumference.

**Table 2:** Demographic, socioeconomic and geographic characteristics of the included participants at baseline (CHARLS 2011).

Characteristics	Overall	Urban	Rural	*p* ^†^
	(*n* = 7397)	(*n* = 2418)	(*n* = 4979)	
**Age group**				0.064
45–49 years	1437 (19.4%)	502 (20.8%)	935 (18.8%)	
50–59 years	2641 (35.7%)	880 (36.4%)	1761 (35.4%)	
60–69 years	2182 (29.5%)	675 (27.9%)	1507 (30.3%)	
≥70 years	1137 (15.4%)	361 (14.9%)	776 (15.6%)	
**Sex**				0.103
Male	3447 (46.6%)	1094 (45.2%)	2353 (47.3%)	
Female	3950 (53.4%)	1324 (54.8%)	2626 (52.7%)	
**Education ^‡^**				<0.001
Illiterate	2173 (29.4%)	462 (19.1%)	1711 (34.4%)	
Literate	1403 (19.0%)	419 (17.3%)	984 (19.8%)	
Primary education	1661 (22.5%)	512 (21.2%)	1149 (23.1%)	
Middle or higher education	2159 (29.2%)	1025 (42.4%)	1134 (22.8%)	
**Marital status**				0.305
Married or cohabiting	6518 (88.1%)	2144 (88.7%)	4374 (87.8%)	
Single	879 (11.9%)	274 (11.3%)	605 (12.2%)	
**Ln(PCE) by setting**				<0.001
Bottom tertile	2167 (29.3%)	478 (19.8%)	1689 (33.9%)	
Middle tertile	3068 (41.5%)	1055 (43.6%)	2013 (40.4%)	
Top tertile	2162 (29.2%)	885 (36.6%)	1277 (25.6%)	
**Region**				<0.001
North China	950 (12.8%)	280 (11.6%)	670 (13.5%)	
Northeast China	503 (6.9%)	200 (8.3%)	303 (6.1%)	
East China	2221 (30.0%)	750 (31.0%)	1471 (29.5%)	
Southcentral China	1747 (23.6%)	647 (26.8%)	1100 (22.1%)	
Southwest China	1366 (18.5%)	417 (17.2%)	949 (19.1%)	
Northwest China	610 (8.2%)	124 (5.1%)	486 (9.8%)	
**HTW phenotype**				<0.001
NTNW	3839 (51.9%)	1070 (44.3%)	2769 (55.6%)	
NTEW/ETNW	2600 (35.1%)	942 (39.0%)	1658 (33.3%)	
ETEW	958 (13.0%)	406 (16.8%)	552 (11.1%)	
**Obesity ^‡^**				<0.001
Normal	3657 (50.0%)	979 (40.8%)	2678 (54.4%)	
Overweight	2748 (37.6%)	1026 (42.8%)	1722 (35.0%)	
Obesity	913 (12.5%)	392 (16.4%)	521 (10.6%)	
**Smoking ^‡^**				0.001
Non-smoker	4506 (61.1%)	1527 (63.4%)	2979 (60.0%)	
Smoker	2869 (38.9%)	883 (36.6%)	1986 (40.0%)	
**Alcohol drinking ^‡^**				0.030
Non-drinker	5089 (68.8%)	1698 (70.3%)	3391 (68.1%)	
Drinker	2304 (31.2%)	717 (29.7%)	1587 (31.9%)	

Note: Data were presented as *n* (%); ^†^ comparison between urban and rural areas; NTNW, normal TG level and normal WC; NTEW, normal TG level and enlarged WC; ETNW, elevated TG level and normal WC; ETEW, elevated TG level and enlarged WC; ^‡^ data for some participants were missing; PCE, per capita expenditures.

**Table 3:** Risk of incident T2DM by HTW phenotype in middle-aged and older Chinese, CHARLS 2011–2018.

Model	HTW Phenotype			*p* ^#^	*p* *
	NTNW	NTEW/ETNW	ETEW		
	(*n* = 3839)	(*n* = 2600)	(*n* = 958)		
**New-onset** **T2DM**	309 (8.05%)	351 (13.50%)	189 (19.73%)		
**Overall**					
Unadjusted	1.00 (reference)	1.69 (1.45, 1.97)	2.58 (2.15, 3.10)	<0.001	<0.001
Model 1	1.00 (reference)	1.68 (1.44, 1.97)	2.54 (2.11, 3.07)	<0.001	<0.001
Model 2	1.00 (reference)	1.39 (1.16, 1.66)	1.88 (1.50, 2.36)	0.000	<0.001
Model 3	1.00 (reference)	1.28 (1.06, 1.54)	1.61 (1.26, 2.06)	0.009	<0.001
**Urban**					
Unadjusted	1.00 (reference)	1.73 (1.28, 2.33)	2.38 (1.70, 3.34)	0.000	<0.001
Model 1	1.00 (reference)	1.66 (1.23, 2.24)	2.20 (1.56, 3.10)	0.001	<0.001
Model 2	1.00 (reference)	1.35 (0.95, 1.92)	1.68 (1.10, 2.57)	0.094	0.017
Model 3	1.00 (reference)	1.23 (0.86, 1.75)	1.36 (0.86, 2.15)	0.268	0.183
**Rural**					
Unadjusted	1.00 (reference)	1.72 (1.44, 2.06)	2.80 (2.25, 3.48)	<0.001	<0.001
Model 1	1.00 (reference)	1.74 (1.45, 2.09)	2.84 (2.27, 3.55)	<0.001	<0.001
Model 2	1.00 (reference)	1.41 (1.14, 1.75)	2.04 (1.55, 2.67)	0.001	<0.001
Model 3	1.00 (reference)	1.31 (1.05, 1.63)	1.76 (1.31, 2.36)	0.015	<0.001

Note: Data were presented as *n* (incidence in %) or hazard ratio (95% CI); Model 1: adjusted for age and sex; Model 2: additionally adjusted for education, marital status, ln(PCE) by setting, region, obesity, smoking, and drinking based on Model 1; Model 3: additionally adjusted for SBP, DBP, TC, HDL-C and LDL-C based on Model 2. NTNW, normal TG level and normal WC; NTEW, normal TG level and enlarged WC; ETNW, elevated TG level and normal WC; ETEW, elevated TG level and enlarged WC; *p* ^#^ for NTEW/ETNW compared to NTNW; *p* * for ETEW compared to NTNW.

## Data Availability

Data were extracted from the China Health and Retirement Longitudinal Study (CHARLS, available at http://charls.pku.edu.cn/en, accessed on 1 August 2020).

## Supplementary Materials

The following are available online at https://www.mdpi.com/article/10.3390/ijerph18073664/s1, Supplementary Figure S1. The six geographic regions in China; Supplementary Table S1. Investigated provinces in CHARLS, Supplementary Table S2. Comparison of general characteristics between the included and excluded subjects in CHARLS 2011; Supplementary Table S3. Associated factors of incident T2DM by multivariable Cox frailty models; Supplementary Table S4. Demographic, socioeconomic and geographic characteristics of the nine transition groups (A-I, CHARLS 2011–2015).
