# Supplementary material for: Transition of Hypertriglyceridemic-Waist Phenotypes and the Risk of Type 2 Diabetes Mellitus among Middle-Aged and Older Chinese: A National Cohort Study
Source: Int J Environ Res Public Health. 2021 Apr 1;18(7):3664. doi: 10.3390/ijerph18073664 (PMC8037185; doi:10.3390/ijerph18073664)
Supplement: Supplementary file 1 [file ijerph-18-03664-s001.pdf]

Transition of hypertriglyceridemic–waist phenotypes and the risk of type 2 diabetes mellitus among middle-aged and older Chinese: a national cohort study

Supplementary Figure S1. The six geographic regions in China

Supplementary Table S1. Investigated provinces in CHARLS

Supplementary Table S2. Comparison of general characteristics between the included and excluded subjects in CHARLS 2011

Supplementary Table S3. Associated factors of incident T2DM by multivariable Cox frailty models

Supplementary Table S4. Demographic, socioeconomic and geographic characteristics of the nine transition groups (A-I, CHARLS 2011-2015)

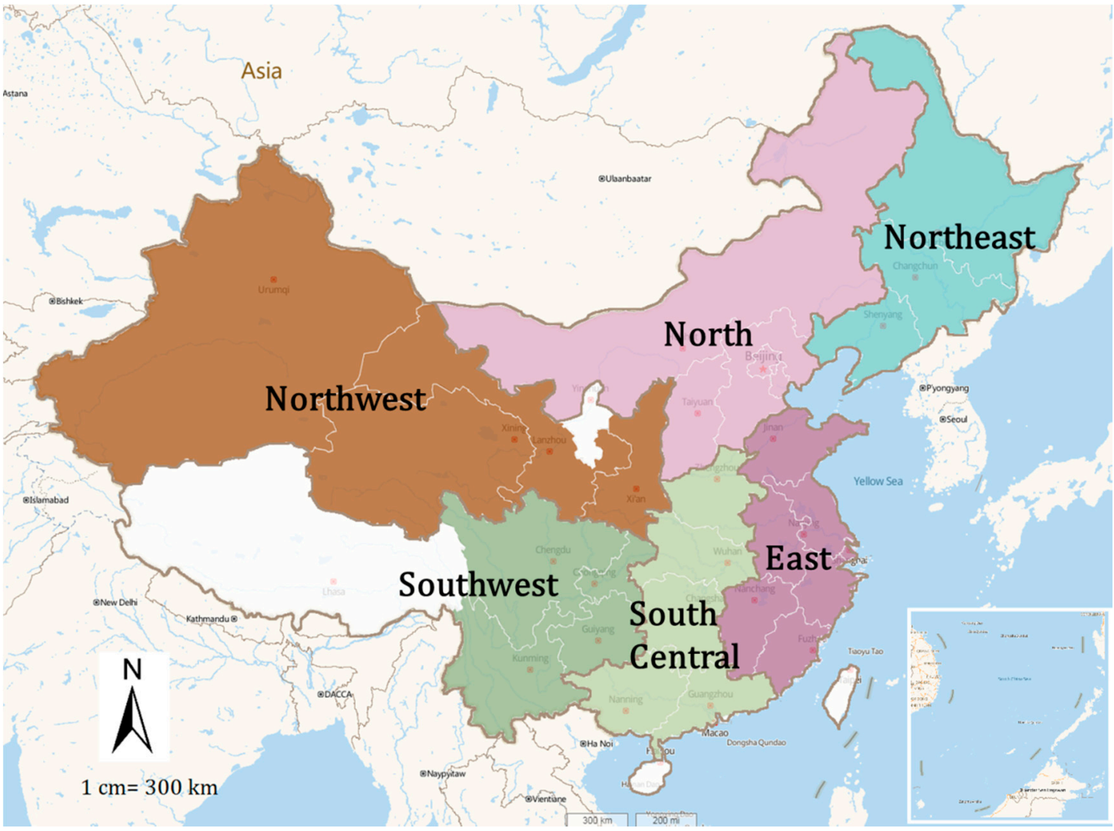

Supplementary Figure S1. The six geographic regions in China

Note: Twenty-eight provinces in Mainland China were randomly chosen in CHARLS except Hainan province, Ningxia Hui Autonomous Region and Tibet province, which were marked in white.

Supplementary Table S1. Investigated provinces in CHARLS

Region	Included provinces
North	Beijing Municipality, Hebei province, Inner Mongolia Autonomous Region, Shanxi province, Tianjin Municipality;
Northeast	Heilongjiang province, Jilin province, Liaoning province;
East	Anhui province, Fujian province, Jiangsu province, Jiangxi province, Shandong province, Shanghai Municipality, Zhejiang province;
South Central	Guangdong province, Guangxi Zhuang Autonomous Region, Hainan province, Henan province, Hubei province, Hunan province;
Southwest	Chongqing Municipality, Guizhou province, Sichuan province, Tibet Autonomous Region, Yunnan province;
Northwest	Gansu province, Ningxia Hui Autonomous Region, Qinghai province, Shaanxi province, Xinjiang Uyghur Autonomous Region;

Supplementary Table S2. Comparison of general characteristics between the included and excluded subjects in CHARLS 2011

Characteristic	Sample (n=17,311)	Excluded subjects (n=9,914)	Included subjects (n=7,397)	*P*‡
**Age group**				<0.001
45-49 years	3496 (20.2%)	2059 (20.8%)	1437 (19.4%)	
50-59 years	6128 (35.4%)	3487 (35.2%)	2641 (35.7%)	
60-69 years	4756 (27.5%)	2574 (26.0%)	2182 (29.5%)	
≥70 years	2931 (16.9%)	1794 (18.1%)	1137 (15.4%)	
**Sex**				<0.001
Male	8419 (48.7%)	4972 (50.2%)	3447 (46.6%)	
Female	8880 (51.3%)	4930 (49.8%)	3950 (53.4%)	
**Education**				<0.001
Illiterate	4733 (27.4%)	2560 (25.9%)	2173 (29.4%)	
Literate	3073 (17.8%)	1670 (16.9%)	1403 (19.0%)	
Primary education	3696 (21.4%)	2035 (20.6%)	1661 (22.5%)	
Middle or higher education	5778 (33.4%)	3619 (36.6%)	2159 (29.2%)	
**Marital status**				0.001
Married or cohabiting	15079 (87.2%)	8561 (86.5%)	6518 (88.1%)	
Single	2220 (12.8%)	1341 (13.5%)	879 (11.9%)	
**Rural/Urban**				<0.001
Rural	10305 (59.5%)	5326 (53.7%)	4979 (67.3%)	
Urban	7006 (40.5%)	4588 (46.3%)	2418 (32.7%)	
**Ln (PCE) by setting**				<0.001
Bottom tertile	4403 (25.4%)	2236 (22.6%)	2167 (29.3%)	
Middle tertile	7298 (42.2%)	4230 (42.7%)	3068 (41.5%)	
Top tertile	5610 (32.4%)	3448 (34.8%)	2162 (29.2%)	
**Region**				<0.001
North China	2350 (13.6%)	1400 (14.1%)	950 (12.8%)	
Northeast China	1288 (7.4%)	785 (7.9%)	503 (6.8%)	
East China	5243 (30.3%)	3022 (30.5%)	2221 (30.0%)	
Southcentral China	4252 (24.6%)	2505 (25.3%)	1747 (23.6%)	
Southwest China	2945 (17.0%)	1579 (15.9%)	1366 (18.5%)	
Northwest China	1233 (7.1%)	623 (6.3%)	610 (8.2%)	
**Obesity**				<0.001
Normal	6449 (48.4%)	2792 (46.5%)	3657 (50.0%)	
Overweight	5028 (37.7%)	2280 (38.0%)	2748 (37.6%)	
Obesity	1845 (13.8%)	932 (15.5%)	913 (12.5%)	
**Smoking**				0.006
Non-smoker	10296 (62.3%)	5790 (63.2%)	4506 (61.1%)	
Smoker	6240 (37.7%)	3371 (36.8%)	2869 (38.9%)	
**Alcohol drinking**				0.577
Non-drinker	11778 (68.6%)	6689 (68.4%)	5089 (68.8%)	
Drinker	5389 (31.4%)	3085 (31.6%)	2304 (31.2%)	

Note: Data were presented as n (%); P‡, comparison between included and excluded subjects; PCE, per capita expenditures.

Supplementary Table S3. Associated factors of incident T2DM by multivariable Cox frailty models

Characteristics	T2DM		
	Model 1	Model 2	Model 3
**Age group**			
45-49 years	1.00 (reference)	1.00 (reference)	1.00 (reference)
50-59 years	1.43 (1.15, 1.76)*	1.40 (1.13, 1.74)*	1.36 (1.09, 1.70) *
60-69 years	1.72 (1.39, 2.14)*	1.64 (1.31, 2.05)*	1.59 (1.26, 2.01) *
≥70 years	1.66 (1.29, 2.13)*	1.57 (1.19, 2.08)*	1.56 (1.17, 2.10) *
**Sex**			
Male	1.00 (reference)	1.00 (reference)	1.00 (reference)
Female	1.08 (0.94, 1.24)	1.08 (0.87, 1.33)	1.11 (0.89, 1.38)
**Education**			
Illiterate	NA	1.00 (reference)	1.00 (reference)
Literate	NA	0.98 (0.80, 1.20)	0.98 (0.80, 1.21)
Primary education	NA	0.91 (0.74, 1.11)	0.91 (0.74, 1.12)
Middle or higher education	NA	0.79 (0.64, 0.98)*	0.80 (0.64, 0.99) *
**Marital status**			
Married or cohabiting	NA	1.00 (reference)	1.00 (reference)
Single	NA	1.12 (0.90, 1.38)	1.13 (0.91, 1.41)
**Ln(PCE) by setting**			
Bottom tertile	NA	1.00 (reference)	1.00 (reference)
Middle tertile	NA	1.00 (0.85, 1.18)	1.01 (0.86, 1.19)
Top tertile	NA	0.98 (0.81, 1.18)	0.99 (0.82, 1.20)
**Region**			
North China	NA	1.00 (reference)	1.00 (reference)
Northeast China	NA	0.54 (0.36, 0.81)*	0.54 (0.36, 0.81) *
East China	NA	0.78 (0.60, 0.99)*	0.78 (0.61, 1.01)
Southcentral China	NA	0.85 (0.66, 1.10)	0.85 (0.65, 1.10)
Southwest China	NA	0.72 (0.54, 0.97)*	0.72 (0.53, 0.96) *
Northwest China	NA	0.88 (0.63, 1.25)	0.89 (0.62, 1.26)
**Obesity**			
Normal	NA	1.00 (reference)	1.00 (reference)
Overweight	NA	1.30 (1.09, 1.55)*	1.27 (1.06, 1.51) *
Obesity	NA	1.81 (1.43, 2.28)*	1.69 (1.34, 2.14) *
**Smoking**			
Non-smoker	NA	1.00 (reference)	1.00 (reference)
Smoker	NA	1.11 (0.91, 1.36)	1.09 (0.89, 1.33)
**Alcohol drinking**			
Non-drinker	NA	1.00 (reference)	1.00 (reference)
Drinker	NA	0.99 (0.83, 1.18)	1.00 (0.83, 1.20)
**Blood pressure**			
SBP	NA	NA	1.00 (reference)
DBP	NA	NA	1.01 (1.00, 1.01)
**Total Cholesterol**			
≤200mg/dL	NA	NA	1.00 (reference)
>200mg/dL	NA	NA	1.21 (1.04, 1.42) *

**HDL-C**			
≥50mg/dL	NA	NA	1.00 (reference)
<50mg/dL	NA	NA	1.24 (1.06, 1.45) *
**LDL-C**			
≤100mg/dL	NA	NA	1.00 (reference)
>100mg/dL	NA	NA	0.98 (0.84, 1.14)

Note: Data were presented as hazard ratios (95% CI); Associations between hypertriglyceridemic–waist and T2DM were assessed using multivariable Cox frailty models with random intercepts to account for clustering of participants by city.

Model 1: adjusted for age and sex. Model 2: additionally adjusted for education, marital status, ln (PCE) by setting, region, obesity, smoking, and drinking based on Model 1. Model 3: additionally adjusted for SBP, DBP, TC, HDL-C and LDL-C based on Model 2.

*T2DM, type 2 diabetes mellitus; PCE, per capita expenditures; SBP, systolic blood pressure, DBP, diastolic blood pressure; HDL-C, high-density lipoprotein cholesterol; LDL-C, low-density lipoprotein cholesterol; NA, not available; * , P<0.05.*

Supplementary Table S4 Demographic, socioeconomic and geographic characteristics of the nine transition groups (A-I, CHARLS 2011-2015)

Characteristic	*A* (N=1768)	*B* (N=636)	*C* (N=131)	*D* (N=375)	*E* (N=976)	*F* (N=436)	*G* (N=25)	*H* (N=229)	*I* (N=414)
**Age group**									
N (Missing Data)	1768 (0)	636 (0)	131 (0)	375 (0)	976 (0)	436 (0)	25 (0)	229 (0)	414 (0)
45-49 years	280 (15.8%)	141 (22.2%)	36 (27.5%)	59 (15.7%)	201 (20.6%)	115 (26.4%)	2 (8.0%)	45 (19.7%)	76 (18.4%)
50-59 years	606 (34.3%)	249 (39.2%)	54 (41.2%)	140 (37.3%)	346 (35.5%)	154 (35.3%)	9 (36.0%)	87 (38.0%)	178 (43.0%)
60-69 years	597 (33.8%)	178 (28.0%)	33 (25.2%)	122 (32.5%)	299 (30.6%)	120 (27.5%)	12 (48.0%)	77 (33.6%)	123 (29.7%)
≥70 years	285 (16.1%)	68 (10.7%)	8 (6.1%)	54 (14.4%)	130 (13.3%)	47 (10.8%)	2 (8.0%)	20 (8.7%)	37 (8.9%)
**Sex**									
N (Missing Data)	1768 (0)	636 (0)	131 (0)	375 (0)	976 (0)	436 (0)	25 (0)	229 (0)	414 (0)
Male	1091 (61.7%)	295 (46.4%)	42 (32.1%)	177 (47.2%)	347 (35.6%)	136 (31.2%)	8 (32.0%)	69 (30.1%)	136 (32.9%)
Female	677 (38.3%)	341 (53.6%)	89 (67.9%)	198 (52.8%)	629 (64.4%)	300 (68.8%)	17 (68.0%)	160 (69.9%)	278 (67.1%)
**Education**									
N (Missing Data)	1768 (0)	636 (0)	131 (0)	375 (0)	976 (0)	436 (0)	25 (0)	229 (0)	414 (0)
Illiterate	472 (26.7%)	183 (28.8%)	39 (29.8%)	120 (32.0%)	305 (31.3%)	125 (28.7%)	10 (40.0%)	74 (32.3%)	106 (25.6%)
Literate	388 (21.9%)	109 (17.1%)	19 (14.5%)	72 (19.2%)	169 (17.3%)	65 (14.9%)	6 (24.0%)	36 (15.7%)	81 (19.6%)
Primary education	413 (23.4%)	159 (25.0%)	23 (17.6%)	90 (24.0%)	218 (22.4%)	98 (22.5%)	2 (8.0%)	57 (24.9%)	103 (24.9%)
Middle or higher education	495 (28.0%)	185 (29.1%)	50 (38.2%)	93 (24.8%)	283 (29.0%)	148 (33.9%)	7 (28.0%)	62 (27.1%)	124 (30.0%)
**Marital status**									
N (Missing Data)	1768 (0)	636 (0)	131 (0)	375 (0)	976 (0)	436 (0)	25 (0)	229 (0)	414 (0)
Married or cohabiting	1562 (88.3%)	569 (89.5%)	119 (90.8%)	330 (88.0%)	869 (89.0%)	398 (91.3%)	21 (84.0%)	208 (90.8%)	380 (91.8%)
Single	206 (11.7%)	67 (10.5%)	12 (9.2%)	45 (12.0%)	107 (11.0%)	38 (8.7%)	4 (16.0%)	21 (9.2%)	34 (8.2%)
**Rural/Urban**									
N (Missing Data)	1768 (0)	636 (0)	131 (0)	375 (0)	976 (0)	436 (0)	25 (0)	229 (0)	414 (0)
Rural	1323 (74.8%)	456 (71.7%)	81 (61.8%)	278 (74.1%)	619 (63.4%)	275 (63.1%)	12 (48.0%)	139 (60.7%)	243 (58.7%)
Urban	445 (25.2%)	180 (28.3%)	50 (38.2%)	97 (25.9%)	357 (36.6%)	161 (36.9%)	13 (52.0%)	90 (39.3%)	171 (41.3%)
**Ln(PCE) by setting**									
N (Missing Data)	1768 (0)	636 (0)	131 (0)	375 (0)	976 (0)	436 (0)	25 (0)	229 (0)	414 (0)
Bottom tertile	591 (33.4%)	198 (31.1%)	38 (29.0%)	108 (28.8%)	290 (29.7%)	133 (30.5%)	5 (20.0%)	61 (26.6%)	103 (24.9%)
Middle tertile	752 (42.5%)	268 (42.1%)	48 (36.6%)	154 (41.1%)	387 (39.7%)	187 (42.9%)	12 (48.0%)	94 (41.0%)	173 (41.8%)
Top tertile	425 (24.0%)	170 (26.7%)	45 (34.4%)	113 (30.1%)	299 (30.6%)	116 (26.6%)	8 (32.0%)	74 (32.3%)	138 (33.3%)
**Region**									
N (Missing Data)	1768 (0)	636 (0)	131 (0)	375 (0)	976 (0)	436 (0)	25 (0)	229 (0)	414 (0)
North China	156 (8.8%)	75 (11.8%)	30 (22.9%)	46 (12.3%)	149 (15.3%)	71 (16.3%)	3 (12.0%)	38 (16.6%)	76 (18.4%)
Northeast China	106 (6.0%)	41 (6.4%)	16 (12.2%)	20 (5.3%)	61 (6.3%)	34 (7.8%)	3 (12.0%)	22 (9.6%)	36 (8.7%)

East China	584 (33.0%)	194 (30.5%)	30 (22.9%)	117 (31.2%)	340 (34.8%)	131 (30.0%)	9 (36.0%)	85 (37.1%)	126 (30.4%)
Southcentral China	427 (24.2%)	140 (22.0%)	18 (13.7%)	91 (24.3%)	226 (23.2%)	99 (22.7%)	7 (28.0%)	43 (18.8%)	104 (25.1%)
Southwest China	332 (18.8%)	118 (18.6%)	23 (17.6%)	59 (15.7%)	112 (11.5%)	76 (17.4%)	3 (12.0%)	28 (12.2%)	41 (9.9%)
Northwest China	163 (9.2%)	68 (10.7%)	14 (10.7%)	42 (11.2%)	88 (9.0%)	25 (5.7%)	0 (0.0%)	13 (5.7%)	31 (7.5%)
**Obesity**									
N (Missing Data)	1755 (13)	629 (7)	129 (2)	372 (3)	968 (8)	434 (2)	25 (0)	228 (1)	412 (2)
Normal	1453 (82.8%)	366 (58.2%)	49 (38.0%)	210 (56.5%)	214 (22.1%)	73 (16.8%)	5 (20.0%)	20 (8.8%)	22 (5.3%)
Overweight	295 (16.8%)	253 (40.2%)	76 (58.9%)	149 (40.1%)	523 (54.0%)	231 (53.2%)	19 (76.0%)	123 (53.9%)	208 (50.5%)
Obesity	7 (0.4%)	10 (1.6%)	4 (3.1%)	13 (3.5%)	231 (23.9%)	130 (30.0%)	1 (4.0%)	85 (37.3%)	182 (44.2%)
**Smoking**									
N (Missing Data)	1763 (5)	634 (2)	131 (0)	375 (0)	972 (4)	435 (1)	24 (1)	229 (0)	413 (1)
Non-smoker	876 (49.7%)	390 (61.5%)	90 (68.7%)	231 (61.6%)	695 (71.5%)	310 (71.3%)	17 (70.8%)	166 (72.5%)	294 (71.2%)
Smoker	887 (50.3%)	244 (38.5%)	41 (31.3%)	144 (38.4%)	277 (28.5%)	125 (28.7%)	7 (29.2%)	63 (27.5%)	119 (28.8%)
**Alcohol drinking**									
N (Missing Data)	1767 (1)	636 (0)	131 (0)	375 (0)	975 (1)	435 (1)	25 (0)	229 (0)	414 (0)
Non-drinker	1103 (62.4%)	441 (69.3%)	104 (79.4%)	266 (70.9%)	728 (74.7%)	316 (72.6%)	17 (68.0%)	173 (75.5%)	309 (74.6%)
Drinker	664 (37.6%)	195 (30.7%)	27 (20.6%)	109 (29.1%)	247 (25.3%)	119 (27.4%)	8 (32.0%)	56 (24.5%)	105 (25.4%)

Note: Data were presented as n (%); PCE, per capita expenditures; the definitions of group A to I were listed as follows:

Group A, the consistent phenotype of NTNW;

Group B, NTNW to NTEW/ETNW;

Group C, NTNW to ETEW;

Group E, the consistent phenotype of NTEW/ETNW;

Group D, NTEW/ETNW to NTNW;

Group F, NTEW/ETNW to ETEW;

Group I, the consistent phenotype of ETEW;

Group G, ETEW to NTNW;

Group H, ETEW to NTEW/ETNW.
